# Sika deer velvet antler protein extract modulater bone metabolism and the structure of gut microbiota in ovariectomized mice

**DOI:** 10.1002/fsn3.3316

**Published:** 2023-03-13

**Authors:** Wang Pan, Juan Du, Liping An, Guangyu Xu, Guangxin Yuan, Yu Sheng, Jingbo Sun, Manli Wang, Nanxi Zhao, Xiao Guo, Hongyu Li, Xiao Han

**Affiliations:** ^1^ Department of Pharmaceutical Analysis Beihua University College of Pharmacy Jilin China

**Keywords:** gut microbiota, osteoporosis, OVX mice, protein extract, sika deer velvet antler

## Abstract

Osteoporosis is a systemic osteopathy characterized by bone metabolism disorders that become more serious with age increases in postmenopausal women. Recent studies have found that antler protein is the main bioactive component of cervus pantotrichum, and it has a positive regulatory effect on bone metabolism and can improve estrogen level. This study aimed to investigate the effect of velvet antler extract (VAE) on the prevention of osteoporosis and the modulation of gut microbiota in ovariectomized (OVX) mice. OVX mice treated with 12 weeks of VAE exhibited higher levels of serum BGP, Ca^2+^, CT, and HyP (*p* < .05). Micro‐CT scans showed that VAE significantly elevated bone volume fraction (BV/TV), trabecular bone number (Tb.N), trabecular bone thickness (Tb.Th), trabecular bone connection density (Conn.D), decreased trabecular separation (Tb.Sp), and structural modality index (SMI) than untreated OVX mice. The right tibial retinaculum in the VAE group was clearer, with a clearer reticular structure, smaller gaps, a tighter distribution, and a more orderly arrangement. The gut microbiota of the cecal contents was analyzed by 16 s rDNA amplicon sequencing. The data indicated that VAE modulated the species, numbers, and diversity of the gut microbiota in OVX mice. Ovariectomy caused dysbiosis of the intestinal microbiota by increasing the ratio of *Firmicutes* to *Bacteroidetes* in mice, but the ratio decreased after treatment with VAE. These results suggest that VAE has a therapeutic effect on OVX mice via modulate bone‐related biochemical markers in serum and structure of gut microbiota.

## Abstract 2

In this paper, I show that velvet antler extract has a positive effect on osteoporosis model mice. I found that velvet antler extract can improve the serum biochemical indexes, bone microstructure, and intestinal flora of osteoporosis model mice. In addition, I noticed that the antler extract significantly increased the content of Akkermansia bacteria in the intestinal tract of mice, indicating that Akkermansia bacteria may be a new biomarker of osteoporosis.

## 1 INTRODUCTION

Osteoporosis is a systemic osteopathy characterized by bone metabolism disorders that become more serious with age increase in postmenopausal women (Wang, Ding, et al., 2021; Wang, Wang, et al., 2021). The dynamic balance between osteoblasts and osteoclasts is a decisive factor in the development of osteoporosis (Lee et al., 2022). The factors of primary osteoporosis include old age, lack of sexual steroids, and increased oxidative stress. Estrogen deficiency can lead to changes in gut microbiota (GM), dysfunction of intestinal epithelial barrier, and dysfunction of the immune system, which may promote harmful intestinal metabolites to the entire organism through the intestinal epithelial barrier. It promotes the production of osteoclast‐promoting factors by immune cells, thus promoting osteoclast activation and bone resorption (Liu et al., 2019). Clinical studies have shown that the severity of bone loss correlates with the diversity of the gut microbiome (Das et al., 2019). However, long‐term use of estrogen in postmenopausal women, such as 17‐estradiol, may cause various side effects, such as gastrointestinal tolerance, breast cancer, ovarian cancer, or endometrial cancer. It is still a great challenge to develop safe and effective drugs for osteoporosis treatment and bone tissue regeneration (Barcelos et al., 2021; Janiszewska et al., 2014).

Cervus Pantotrichum protein is the main active component of Cervus Pantotrichum, and the protein omics study of Cervus Pantotrichum has also confirmed that polypeptide is the main bioactive component of Cervus Pantotrichum, and it has a positive regulatory effect on bone metabolism and can improve the estrogen level (Zhang et al., 2021). The sika deer antler preparation has been used to strengthen bones and muscles, promote blood flow, reduce chronic joint pain, lower cholesterol levels, increase immune function, and have a positive impact on antioxidant capacity. Studies have shown that sika deer antler may have preventive and therapeutic effects on femoral head necrosis, and may also help to produce sperm and strengthen bones (Pu & Peng, 2018; Wang et al., 2020). Pilose antlers may be used as a source of calcium to treat bone degeneration and strengthen bone (Chen et al., 2019; Chu et al., 2021). Because many patients have lost bone mass before receiving treatment, it is more necessary to accelerate bone formation to treat osteoporosis.

Previous studies (Ren et al., 2019; Tseng et al., 2016) have found that sika deer antler protein extract can obviously alleviate osteoporosis. In order to further study the mechanism of treating osteoporosis, the mechanism of its action on osteoporosis was clarified by studying the changes of serum biochemical indexes, micro‐CT scanning, and intestinal flora in osteoporosis model mice.

## 2 MATERIALS AND METHODS

### 2.1 Materials

Sika deer antler was purchased from Xiangyang Deer Farm in Jilin (Jilin, China). 12‐week‐old female SPF ICR mice (license number: SCXK(Ji)‐2017‐0015), weighing 25 ± 2 g, were provided by Changchun Yisi Experimental Animal Technology Co., Ltd. (Changchun, China). The experiment follows the Guide to Care and Use of Laboratory Animals. This project was authorized by the Ethics Committee of Beihua University (Approval No. 2019‐069). ELISA kit was purchased from Jiangxi Hailian Biotechnology Co., Ltd. (Jiangxi, China).

### 2.2 Preparation of VAE

0.2 kg velvet antler were cut into small pieces and rinsed with distilled water at 4°C to remove blood. The pieces were ground with a cold acetic acid solution (pH 3.5) using a homogenizer. After homogenization, the mixture was ultrasonically extracted for 30 min in an ice bath, and the supernatant was extracted by centrifugation at 3500 rpm for 15 min at 4°C. Ammonium sulfate was added to the supernatant until its final concentration was 80%, and then centrifuged again at 4°C. The collected precipitate was dissolved in distilled water, and the solution was dialyzed with a dialysis bag with a cut‐off molecular weight of 5 kDa for 48 h. After centrifugation, the supernate was stored at −80°C for at least 12 h, and then got freeze‐dried by the lyophilizer for 48 h to obtain VAE.

### 2.3 Establishment of osteoporosis model in mice and drug intervention

Forty 12‐week‐old female SPF ICR mice were housed at a conventional temperature (22–25°C) with a 12‐hour light/12‐hour dark cycle and unrestricted access to food and water. After one week of adaption, all mice were randomly allocated into four groups (*n* = 10): the blank control group (Con), the model group (Mod), the positive drug group (Pir), and the velvet antler extract group (VAE). The animals were weighed and intraperitoneally injected with 8% uratan (0.1 mL/10 g). Under sterile conditions and anesthesia, a bilateral ovariectomy was dislodged to induce osteoporosis in the mice in the Mod, Pir, and VAE groups. A sham procedure in which only a certain amount of fat was removed without ovariectomy was performed on the Con group mice. Mice in the Pir group intragastrically received Compound Pilose Antler Jiangu Capsule Liquid (1.7 g/kg). Meanwhile, those in the VAE group were given VAE (300 mg/kg) by gavage every day. Others in the Con and Mod groups received an equal dose of physiological saline intragastrically. Each animal received continuous administration for 12 weeks.

### 2.4 Collection and preparation of serum samples, bone tissue samples, and colon contents

Mice were placed in a sealed container filled with ether for 6 minutes to ensure that they can be taken out quickly after coma. Blood samples were acquired by drawing peripheral blood from mice, then centrifuged at 4000 rpm for 15 min to extract serum, which was subsequently stored at −80°C for use.

After collecting serum samples, the mice were sacrificed by cervical dislocation and placed on the ultra‐clean workbench. The colonic contents samples of mice were taken out and put in an aseptic box, then stored at −80°C. The mice's tibias were removed along the direction of the right leg joint. The tibia was preserved in 4% paraformaldehyde for 36 h without any connected muscle or connective tissue.

### 2.5 Determination of BGP, Ca^2+^, CT, E2, FOXP3 and HyP levels in mice serum

The contents of BGP, Ca^2+^, CT, E2, FOXP3, and HyP in serum were measured using ELISA kits, which were used according to the manufacturer's instructions.

### 2.6 Micro‐CT measurements

The Swiss Micro‐CT μ100 scanner (resolution of 100 μm) was used to scan and image tibia samples from mice. The three‐dimensional visualization was accomplished after the samples had been placed in the sample tank and the scanning settings had been set. The microstructure and parameters in the lesion area of mice, including the trabecular number (Tb.N), bone volume (BV/TV), structural mode index (SMI), trabecular thickness (Tb.Th), trabecular connection density (Conn.D), and trabecular separation (Tb.Sp), were measured and analyzed (Jin et al., 2021; Lee et al., 2021; Wang, Ding, et al., 2021; Wang, Wang, et al., 2021; Yang et al., 2022).

### 2.7 16S rDNA sequencing

Total DNA was prepared from the samples of mice colon contents with the QIAamp Fast DNA Stool Mini Kit (Qiagen, Hilden, Germany), and the DNA purity quotient and concentration were determined using a nanodrop 2000 spectrophotometer.

A target fragment library is built using microbial ribosomal RNA that might reflect the target sequences of the makeup and variety of the microbial community (Di et al., 2021). The primers were developed based on the conserved portions of the sequence, and the generic primers were supplemented with universal connectors and sample‐specific barcode sequences. The Trans Gen AP221‐02 reagent was used for PCR reactions of variable regions of specific gene snippets or rRNA genes (V3 and V4). According to previous studies, primers 338F (5′‐ACTCCTACGGGAGGCAGCAG‐3′) and 806R (5′‐GGACTACHVGGGTWTCTAAT‐3′) were used for amplifying the V3‐4 zones (Wen et al., 2020). 2% agarose gel electrophoresis was used to identify the amplified PCR products, which were subsequently recovered and purified. The Axyprep PCR Clean‐up Kit is used for recycling (the optimum insertion range for good sequencing was 200–450 bp). The double‐ended sequencing of 2 × 300 bp was performed on a MiSeq sequencer (600 cycles).

### 2.8 Statistical analysis

The SPSS 19 statistical software was used to process the experimental data. The average value of three experiments was calculated, and the findings were given in the form of^−^x ± s. A one‐way analysis of variance (ANOVA) was utilized to make a comparison between groups. Significant results were those with a *p*‐value of less than .05.

## 3 RESULTS

### 3.1 Effects of VAE on serum biochemical indicators in mice

**FIGURE 1:**
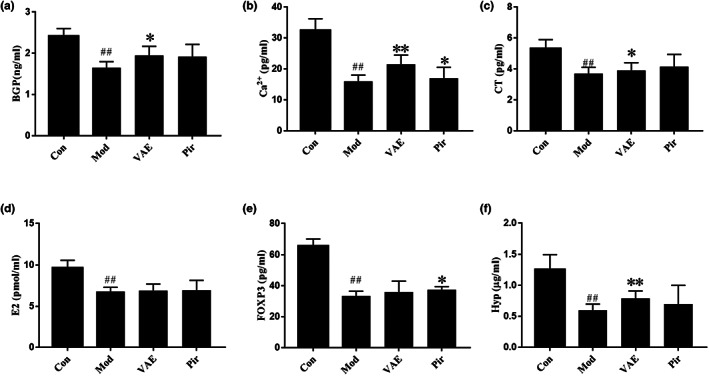
(a–f) Serum biochemical indicators of mice. (a–f: the contents of BGP, Ca^2+^, CT, E2, FOXP3 and Hyp in serum; ^##^ *p* < .01 vs Blank control group; *p < .05 vs Model group; **p < .01 vs Model group. Con, Blank control group; Mod, Model group; VAE, velvet antler extract group; Pir, positive drug group).

Compared with mice in the Con group (Figure 1a–f), there were significantly decreased contents of BGP, Ca^2+^, CT, E2, FOXP3, and HyP in the serum of the Mod group (*p* < .05), indicating the osteoporosis models were successfully and efficiently established in the research.

The levels of serum biochemical parameters such as BGP, Ca^2+^, CT, and HyP were substantially higher in the VAE group (*p* < .05) than in the Mod group, but there was no significant difference in the concentrations of E2 and FOXP3 (*p* > .05). However, in Pir group, only the contents of Ca^2+^ and FOXP3 were significantly different from those in the model group (*p* < .05), but there were no significant differences in BGP, CT, E2, and HyP (*p* > .05).

### 3.2 The right proximal tibia micro‐CT scan results

**FIGURE 2:**
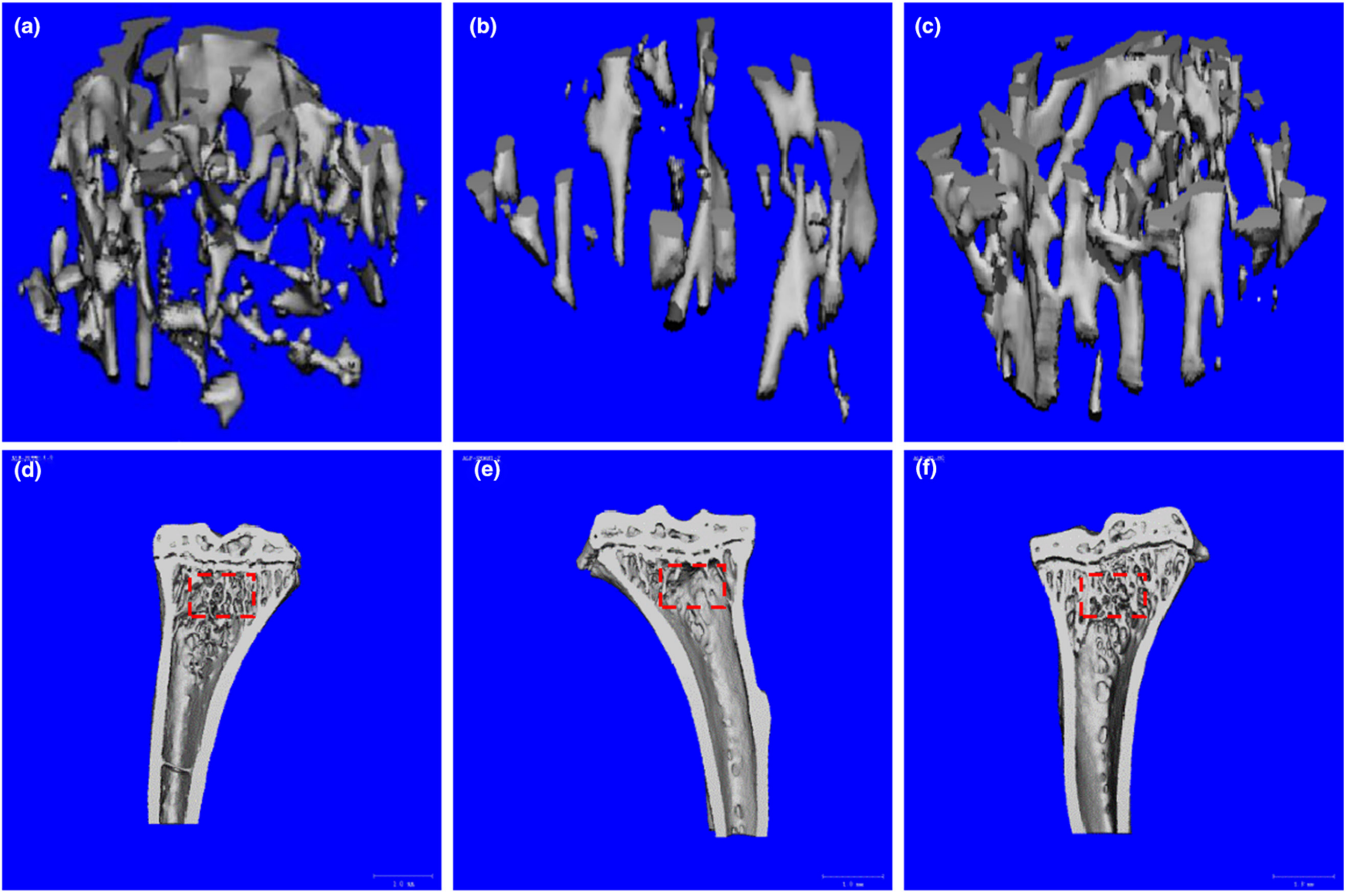
(a–f) 3D reconstruction image of proximal tibia of mice. The red dotted line area represents the quantitative analysis of the histomorphometry (a and d: Con group, b and e: Mod group, c and f: VAE group).

The change of bone trabecular structure is an important factor in osteoporosis. To detect the effect of VAE on bone trabecular structure, the microstructure of bone trabecular was observed by Micro‐CT. The results of Micro‐CT showed that the right tibial trabeculae in the Con group were evenly distributed and closely arranged, with large thickness and small spacing, and the reticular structures were clearer (Figure 2a,d).

Compared with the Con group, the bone trabeculae of the Mod group were significantly thinner, the gap was wider, the number was reduced, the integrity of the morphological structure was poor, sparse, and broken, the connection was not reticular, the bone marrow cavity was significantly enlarged, and blank areas appeared. Compared with the Mod group, the width and the number of tibial trabeculae in the VAE group increased significantly, the fractures of bone trabeculae decreased, and the blank area of the bone marrow cavity became smaller, which was close to the Con group, as shown in Figure 2a–f.

As shown in Table 1, compared with the Con group, bone volume (BV/TV), trabecular thickness (Tb.Th), trabecular number (TB.N), and trabecular connection density (Conn.D) were significantly decreased in the Mod group, but increased significantly after VAE treatment (*p* < .05). In addition, trabecular separation (Tb.Sp) and structural model index (SMI) were significantly decreased in the VAE group (*p* < .05). These results showed that VAE could inhibit tibia bone loss, maintain bone trabecular states, and improve bone biochemical indicators.

**TABLE 1:** Analysis of the sensitive area value of the trabecular bone microstructure of the proximal tibia in mice.

	Con	Mod	VAE
BV/TV	0.29 ± 0.05	0.18 ± 0.06^##^	0.26 ± 0.05**
Tb.N	3.8 ± 0.47	2.71 ± 0.23^#^	3.56 ± 0.21**
Tb.Th	0.14 ± 0.05	0.08 ± 0.01^#^	0.12 ± 0.04*
Tb.Sp	0.32 ± 0.01	0.49 ± 0.02^#^	0.44 ± 0.02**
SMI	2.11 ± 0.16	2.92 ± 0.05^##^	2.70 ± 0.08*
Conn.D	38.99 ± 5.05	23.61 ± 5.76^#^	34.86 ± 4.05*

Abbreviations: Con, Blank control group; Mod, Model group; VAE, velvet antler extract group.

^#^ *p* < .05 vs Blank control group.

^##^ *p* < .01 vs Blank control group.

* *p* < .05 vs Model group.

** *p* < .01 vs Model group.

### 3.3 OTU Venn analysis results

**FIGURE 3:**
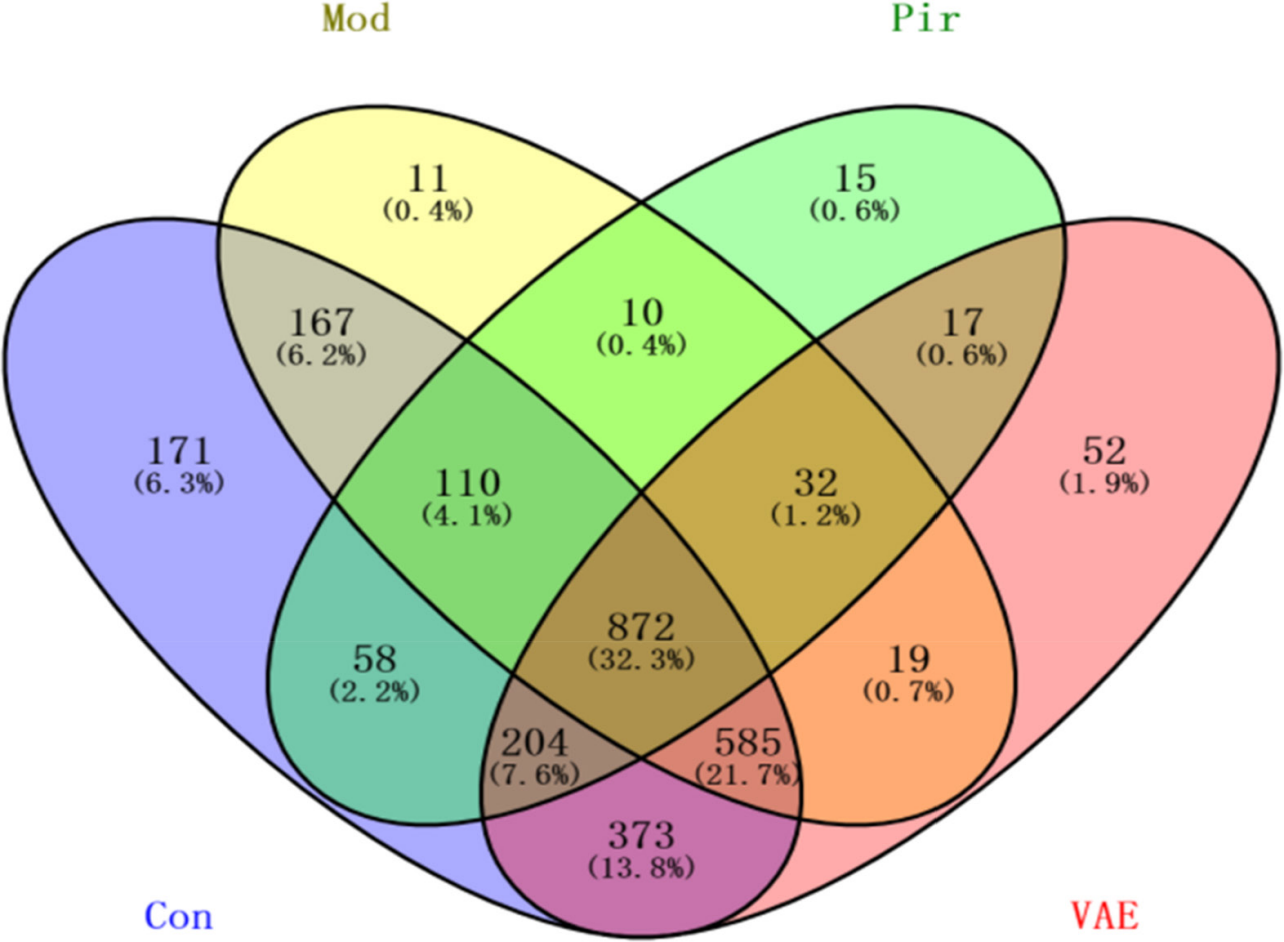
VAE makes the number of intestinal flora OTUs in osteoporotic mice close to the control group. Con, Blank control group; Mod, Model group; Pir, positive drug group; VAE, velvet antler extract group.

In order to improve the efficiency of the analysis, the sequences were clustered according to a certain degree of similarity in microbial diversity analysis, and each formed category is called an OTU. As shown in Figure 3 and Table 2, there were 2540 OTUs in the Con group, 1806 OTUs in the Mod group, 1318 OTUs in the Pir group, and 2154 OTUs in the VAE group, indicating that the OTU species of intestinal flora in the Con group were the most abundant. After ovariectomy, the OTUs of the intestinal flora of the Mod group mice changed drastically, and the species dropped significantly. The OTUs of the intestinal flora were greatly restored in mice treated with VAE, which proved that VAE has the effect of regulating the types of intestinal flora in osteoporotic mice. However, the OTU number of intestinal flora decreased in mice treated with positive drugs.

**TABLE 2:** OTU Venn table.

	Con	Mod	Pir	VAE
OTU number	2540	1806	1318	2154

Abbreviations: Con, Blank control group; Mod, Model group; Pir, positive drug group; VAE, velvet antler extract group.

### 3.4 Alpha diversity analysis results

**FIGURE 4:**
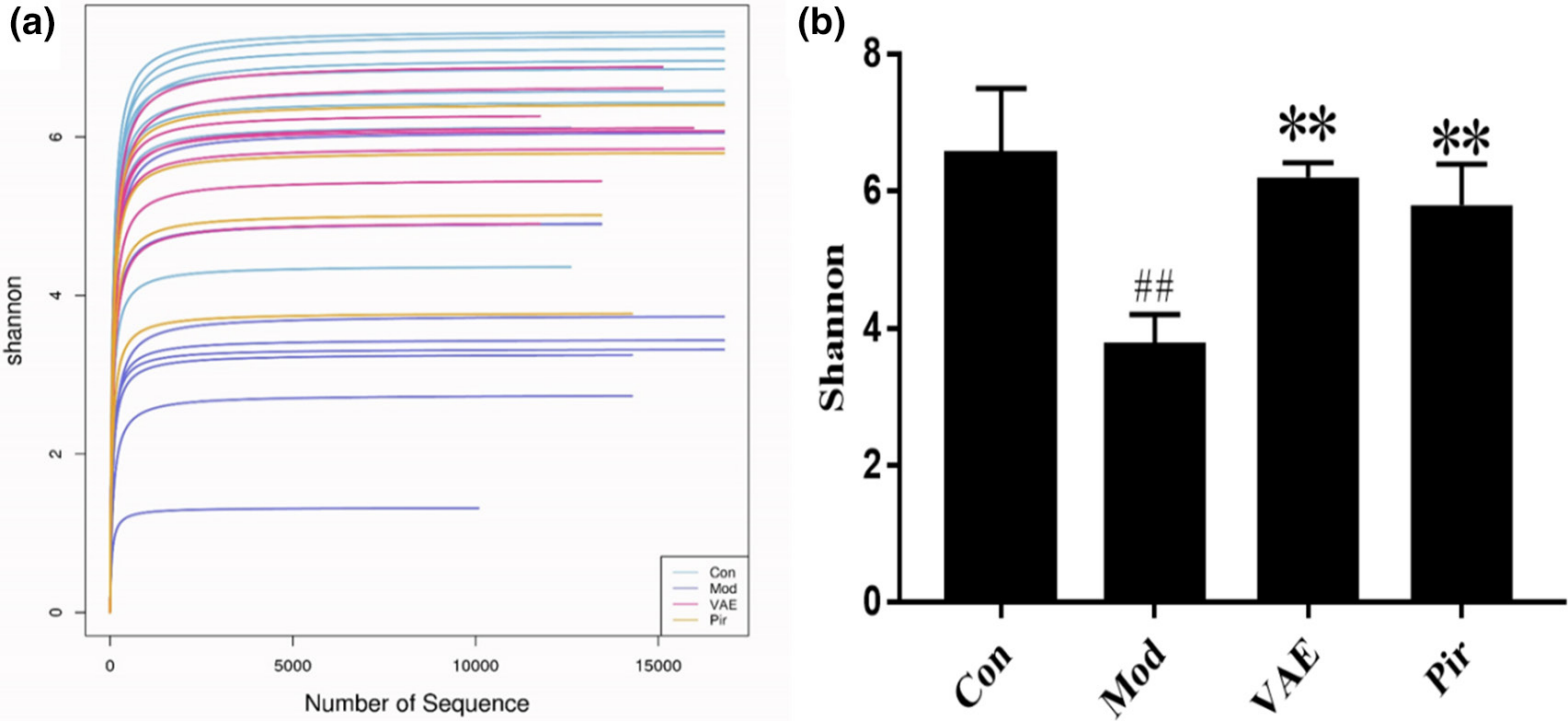
(a) Shannon Diversity Index curves; (b) Shannon diversity indexes. ^##^ *p* < .01 vs Blank control group; **p < .01 vs Model group; Con, Blank control group; Mod, Model group; Pir, positive drug group; VAE, velvet antler extract group.

In order to determine the alpha diversity, the average Shannon index was calculated. This process could fully characterize the diversity of bacterial communities in the sample. The Shannon index reflects the number of species as well as the average or uniformity of the abundance of different species in the sample. The OTU level sparse curve of diversity estimation reached the plateau stage, indicating that the sequencing depth in this experiment was sufficient to explain most bacterial species. The data of the VAE group was higher and more concentrated than that of the Pir and Mod groups, and the higher estimators represented greater diversity, which indicated that the alpha diversity index is positively correlated with BMD (bone mineral density), as shown in Figure 4a. As shown in Figure 4b, the alpha diversity of Mod group mice decreased significantly. After VAE treatment, the alpha diversity almost reached the level of normal mice.

### 3.5 Beta diversity analysis results

PCoA is a visualization method to study similarities or differences in data, through which structural differences between individuals or groups can be observed. Each point corresponds to a different sample, and the same color indicates the same group. The closer the distance between the two points, the smaller the difference in the community composition of the two points.

**FIGURE 5:**
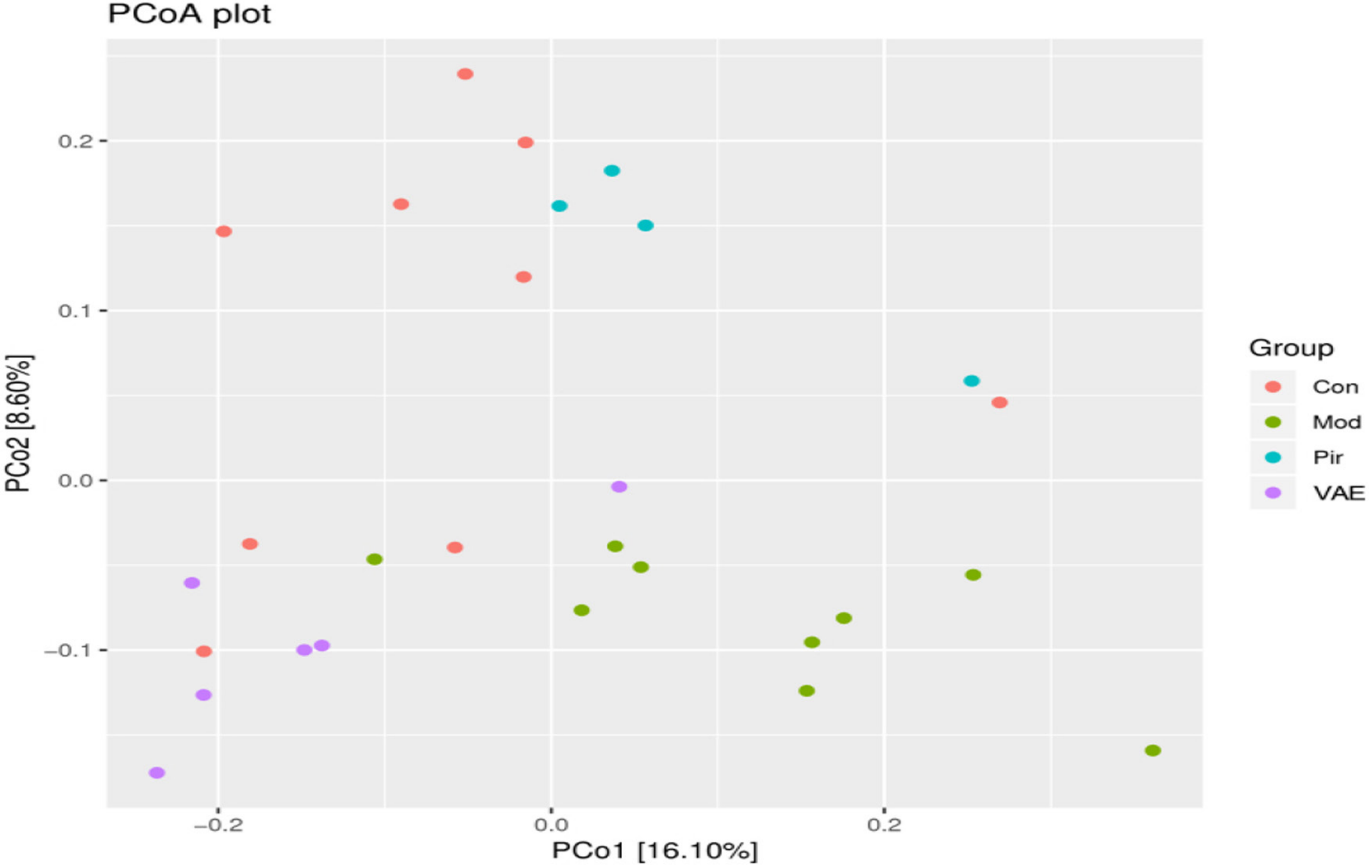
Beta diversity analysis diagram. Con, Blank control group; Mod, Model group; Pir, positive drug group; VAE, velvet antler extract group.

As shown in Figure 5, the contributions of abscissa PCO1 and ordinate PCO2 were 16.1% and 8.6%, respectively, which can effectively distinguish the composition of intestinal‐related microbial communities in mice in each group. Except for individual points, the microbial community composition and distribution of mice in each group were relatively concentrated and could be well distinguished. Among them, the β diversity in the Mod group varies greatly, while the community composition of the VAE group was relatively concentrated and close to that of the Con group on the abscissa PCoA.

### 3.6 Statistical results of species classification

According to the species abundance table and species annotation table, the 20 species with the highest abundance were selected for classification, the relative abundance was calculated, and the histogram of sample abundance comparison was drawn and displayed in the form of a stacked bar chart. The vertical axis represented the relative abundance of different flora.

Based on the results of OTUs, species annotation was carried out for OTUs. The compositions of intestinal microorganisms in the four groups of mice at the genus level were statistically analyzed.

**FIGURE 6:**
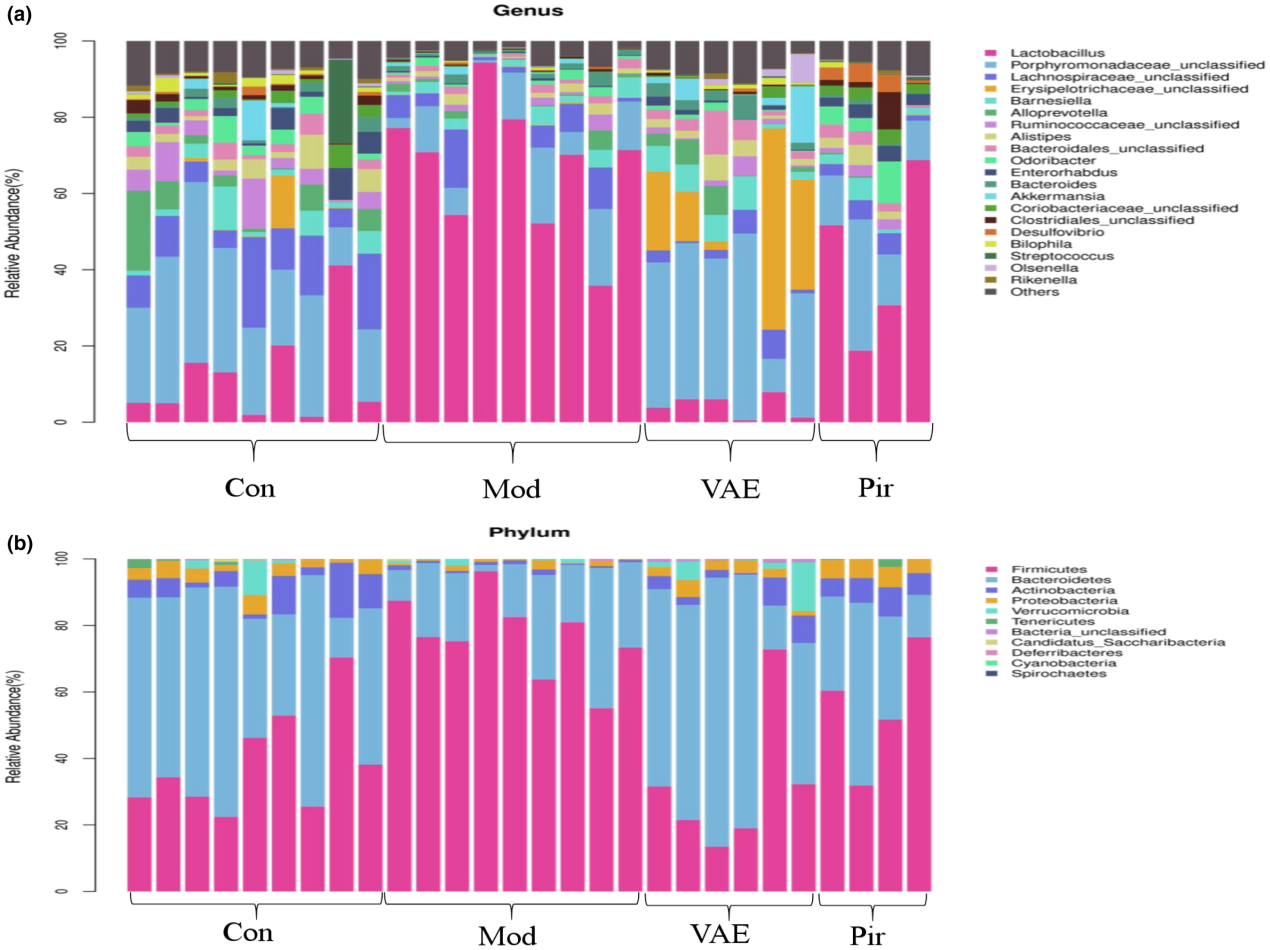
(a) Stacked bar chart of species abundance at different genera taxonomic levels. (b) Stacked bar chart of species abundance at different phyla taxonomic levels. Con, Blank control group; Mod, Model group; Pir, positive drug group; VAE, velvet antler extract group.

At the genus level, a total of 17 genera with proportions higher than 1% were detected, as shown in Figure 6a. *Lactobacillus* and *Porphyromonadaceae* make up the majority of all samples. Compared with the Con group, the abundance of *Lactobacillus* in the Mod group mice increased significantly, while the abundance in the VAE group mice decreased significantly and was lower than that in the Con group. In addition, the abundance of *PorPhyromonadaceae* and *LachnosPiraceae* was significantly lower in the Mod group mice than in the Con group, and the abundance of both bacteria recovered after VAE treatment and approached that of healthy mice. Notably, the abundance of *Akkermansia* bacteria was significantly higher after VAE treatment than in healthy mice. Table 3 indicates that the species and trend of bacteria in the VAE group and Con group mice were almost the same, which proves that VAE can ameliorate osteoporosis, and the diversity of intestinal flora was obviously close to that in the Con group.

**TABLE 3:** Relative species abundance at the genus level of intestinal flora of mice in each group (%).

Genus	Con	Mod	Pir	VAE
Lactobacillus	12.07	67.29	42.44	9.23
Porphyromonadaceae_unclassified	30.47	10.39	17.81	34.41
Lachnospiraceae_unclassified	11.57	5.75	3.74	10.45
Erysipelotrichaceae_unclassified	1.72	0.10	0.10	7.57
Barnesiella	3.84	2.82	2.74	5.49
Alloprevotella	5.27	1.30	0.22	3.15
Ruminococcaceae_unclassified	5.06	1.15	1.74	1.72
Alistipes	3.46	1.47	2.52	2.95
Bacteroidales_unclassified	2.48	1.39	2.42	4.24
Odoribacter	2.99	1.20	4.83	0.82
Enterorhabdus	3.46	0.35	3.24	0.93
Bacteroides	1.51	1.34	0.54	2.84
Akkermansia	1.56	0.52	0.00	3.99
Coriobacteriaceae_unclassified	2.02	0.18	3.07	0.74
Clostridiales_unclassified	1.45	0.33	3.23	0.36
Desulfovibrio	0.56	0.16	3.36	0.20
Bilophila	1.42	0.13	0.36	0.82
Streptococcus	2.59	0.11	0.41	0.18
Olsenella	0.39	0.07	0.13	1.83
Rikenella	0.82	0.00	0.31	0.36
Others	5.31	3.95	6.78	7.73

Abbreviations: Con, Black contral group; Mod, Model group; Pir, positive drug group; VAE, velvet antler extract group.

At the gate level shown in Figure 6b and Table 4, *Firmicutes*, *Bacteroidetes*, *Actinobacteria*, and *Protebacteria* constituted the four main gates in all samples. The abundance of *Firmicutes* in the Mod group was significantly higher than that in the Con group, while the abundance of *Firmicutes* in the VAE group decreased significantly. The abundance of the remaining phyla in the Mod group was significantly lower than that in the Con group, and after VAE treatment, the abundance increased. The proportion of phyla in the VAE group was basically the same as that in the Con group.

**TABLE 4:** Species relative abundance of intestinal microflora in each group (%).

Phylum	Con	Mod	Pir	VAE
Firmicutes	38.50	76.77	55.06	31.73
Bacteroidetes	49.03	20.72	31.78	56.18
Actinobacteria	6.60	0.90	7.04	4.29
Proteobacteria	3.56	0.93	5.41	3.09
Verrucomicrobia	1.56	0.52	0.00	3.99
Tenericutes	0.43	0.00	0.63	0.02
Bacteria_unclassified	0.12	0.03	0.04	0.58
Candidatus_Saccharibacteria	0.14	0.07	0.02	0.03
Deferribacteres	0.03	0.07	0.02	0.09
Cyanobacteria	0.02	0.00	0.00	0.00
Spirochaetes	0.00	0.00	0.00	0.00

Abbreviations: Con, Black contral group; Mod, Model group; Pir, positive drug group; VAE, velvet antler extract group.

## 4 DISCUSSION

Osteoporosis is a prevalent bone disease worldwide. Patients suffer from reduced bone mass and brittle bones, which seriously affect their quality of life and even lead to fractures, which may eventually lead to disability. Deer antler is widely used in Asian medicine for the treatment of various diseases, in particular, Sika deer antler preparations have positive effects on bone or cartilage disorders in clinical observation (Yao et al., 2021). Lei et al. (2022) showed that the exosomes of pilose antler stem cells rejuvenated the aging human mesenchymal stem cells, and had obvious activities of promoting proliferation and inducing differentiation, which could promote the transformation of mesenchymal stem cells into osteogenesis and cartilage. Deer antler peptides also promote osteoblast proliferation, differentiation, and mineralization in vitro via the insulin signaling pathway (Yun et al., 2020). These studies suggest that deer antler has potential bone protective effects and is beneficial to bone health.

E2 is the most important estrogen for maintaining female function and secondary sexual characteristics, preventing bone loss, and improving bone trabeculae morphology (Lee et al., 2019; Shu et al., 2020). The level of E2 after ovariectomy in mice can reflect whether the establishment of mouse osteoporosis model is successful or not. In the present study, the E2 content in blood of OVX mice decreased significantly, the number of bone trabeculae decreased, and the bone trabeculae became thinner and sparsely arranged. This indicated that an animal osteoporosis model (OVX mice) induced by estrogen deficiency was successfully established by ovariectomy. BGP is an active peptide secreted by osteoblasts that can effectively reflect the activity of newly formed osteoblasts in the body. Luo et al. (2021) showed that BGP can be used as a marker to predict the risk of bone fracture in osteoporosis. As the basic component of bone, Ca^2+^ will be lost to a certain extent when osteoporosis occurs. Also, Ca^2+^ can affect the secretion of CT, which can increase bone mass when it has a high affinity for CTRs on the osteoclast membrane (Yu et al., 2021). Therefore, it is necessary to detect the levels of Ca^2+^ and CT. Bone collagen is rich in HyP. Ingestion of collagen to replenish HyP in the body may be one of the mechanisms of the preventive and therapeutic effects of osteoporosis (Black et al., 2015; Qi et al., 2020). The serum biochemical data in the present study showed that the levels of BGP, Ca^2+^, CT, E2, FOXP3, and HyP in the blood of OVX mice were significantly decreased, while VAE significantly increased the levels of BGP, Ca^2+^, CT, and HyP in the blood of OVX mice. By improving the activity of BGP and the absorption and utilization of calcium, blood calcium was reduced and introduced into bone, thus promoting bone formation and increasing bone mass. Stable expression of Foxp3 is a prerequisite for Treg cells to exert osteoprotective effects (Zemmour et al., 2021). VAE elevated the levels of E2 and FOXP3 in osteoporosis model mice in this study, but there was no significant difference. Micro‐CT was used to evaluate the tibial bone structure. The results showed that the bone trabeculae were reduced in OVX mice, but increased and arranged tightly in VAE‐treated mice, indicating that VAE could improve bone microarchitecture and bone health in OVX mice. VAE promotes bone formation and inhibits bone resorption by increasing the levels of bone‐related biochemical indicators in the blood. The effect of VAE on increasing bone mass and improving osteoporosis was achieved by increasing the levels of bone‐related biochemical indexes in the blood, promoting bone formation, and inhibiting bone resorption, which demonstrated the effectiveness of VAE in treating postmenopausal osteoporosis.

Many studies have confirmed a relationship between gut microbiota and osteoporosis. With the increase of age, the gut microbiota becomes more diverse, and an imbalance of the gut microbiota will affect host health and longevity (Kim & Jazwinski, 2018). An imbalance of the gut microbiota alters the alkalinity of the gut and affects calcium and vitamin D absorption (Locantore et al., 2020). Yatsonsky et al. (2019) showed that the gut microbiota alters BMD by affecting vitamin D levels, calcium reabsorption, inflammation, and the immune and endocrine systems in the host. The ability of the gut microbiota to regulate the organism will gradually weaken with age (Ticinesi et al., 2019).

The results of α‐diversity of gut microbiota in this study showed that the diversity of gut flora was increased in the VAE group of mice, while PCoA analysis of β‐diversity could also clearly distinguish the composition of gut flora in the Mod and VAE groups of mice. These data suggested that ovariectomy significantly promotes bone loss and alters intestinal bacterial distribution in mice, and it is hypothesized that bone mass and intestinal microbiota are closely linked. At the phylum level, the content of *Firmicutes* and *Bacteroidetes* bacteria in OVX mice was significantly higher, and at the genus level, 17 genera were identified with proportions exceeding 1%, and *Lactobacillus* was significantly higher, which belongs to the *Bacteroidetes*. These results showed that the composition, structure, and diversity of the gut microbiota of ovariectomized mice were significantly different from those of normal mice, supporting the idea that gut flora is closely related to bone health. The gut flora of VAE‐treated model mice was similar to that of control mice in terms of species composition, abundance, and structure, as well as increasing and decreasing trends, indicating that VAE reduces the difference in gut flora between osteoporotic and healthy mice and restores microbiota composition.

Colonization of the *Firmicutes*, a major phylum in the human gut, may contribute to the activation of osteoblasts and exacerbate inflammation (Yuan et al., 2022). The *Firmicutes* and the *Bacteroidetes* form an important part of the human microbiota, and their abundance ratios are often used as markers of microbial dysbiosis, with increased ratios that may lead to various diseases. The intestinal flora is a network of microbial species interacting in the gut in a state of dynamic equilibrium (Hernandez, 2017). Dysbiosis of the intestinal flora may increase intestinal permeability, prompting a large number of pathogenic bacteria to translocate into the intestine, triggering local and systemic immune‐inflammatory responses in the intestine, activating massive proliferation and differentiation of osteoclast precursor cells, accelerating bone loss, and triggering osteoporosis. The results showed that the relative abundance ratio of the *Firmicutes* to the *Bacteroidetes* increased in OVX mice compared with the Con group, and the ratio decreased after VAE treatment. It can be seen that the removal of ovaries led to an abrupt decrease in estrogen levels, disruption of intestinal flora, destruction of intestinal barrier function, and impact on the skeletal system, while the administration of VAE to mice improved the composition structure of intestinal flora in OVX mice.

Notably, in this study, the relative abundance of *Akkermansia* in the intestine of mice in OVX mice decreased, but significantly increased after treatment with VAE. *Akkermansia* is a mucinolytic bacterium of the intestine, and its abundance is highly correlated with the progression of diseases such as cancer and inflammatory bowel disease (Li et al., 2017; Wang et al., 2017). It has been shown that *Akkermansia* is an important marker of intestinal homeostasis and has important implications for immune and metabolic regulation (Cheng & Xie, 2021; Ottman et al., 2017). We conjectured that VAE achieves the effect of improving bone quality by increasing the abundance of *Akkermansia* in the intestine, decreasing intestinal permeability, maintaining intestinal barrier function, enhancing intestinal mucosal tightness, reducing inflammatory response, and inhibiting bone trabecular loss. However, the mechanism of action of *Akkermansia* in the treatment of osteoporosis still needs further study.

In conclusion, VAE may improve bone microarchitecture in OVX mice by regulating bone‐related biochemical indicators in the blood and adjusting intestinal flora, thus achieving skeletal protection. The shortcoming of this study is that it failed to determine which components of VAE achieved these beneficial effects. Also, the mechanism through which the flora affects bone quality was not studied in detail. The exact mechanism needs to be further investigated at a later date.

## 5 CONCLUSIONS

In this study, changes in serum bone‐related biochemical indices and intestinal flora diversity in OVX mice were investigated. The results showed that VAE could improve osteoporosis by modulating bone‐related biochemical indices, effectively increasing the number and thickness of tibial trabeculae and making them tightly connected, and regulating the intestinal flora.

## CONFLICT OF INTEREST STATEMENT

The authors have no relevant financial or non‐financial interests to disclose.

## Data Availability

The data that support the findings of this study are available from the corresponding author upon reasonable request.
